# Clinical Predictors of Survival in Patients With BRAF^V600^-Mutated Metastatic Melanoma Treated With Combined BRAF and MEK Inhibitors After Immune Checkpoint Inhibitors

**DOI:** 10.1093/oncolo/oyad300

**Published:** 2023-11-16

**Authors:** Adriana M Kahn, Curtis J Perry, Katrina Etts, Harriet Kluger, Mario Sznol

**Affiliations:** Section of Medical Oncology, Yale School of Medicine, New Haven, CT, USA; Section of Medical Oncology, Yale School of Medicine, New Haven, CT, USA; Section of Medical Oncology, Yale School of Medicine, New Haven, CT, USA; Section of Medical Oncology, Yale School of Medicine, New Haven, CT, USA; Section of Medical Oncology, Yale School of Medicine, New Haven, CT, USA

**Keywords:** immunotherapy, targeted therapy, melanoma, survival

## Abstract

Prospective and between trial comparisons indicate that first-line treatment with immune checkpoint inhibitors improves survival outcomes compared to first-line therapy with combined BRAF and MEK inhibitors in metastatic melanoma containing BRAF^V600E/K^ mutations. Long-term outcomes for BRAF/MEK inhibition after progression on immunotherapy have not been reported. Moreover, clinical variables associated with outcome from treatment with combined BRAF/MEK inhibition were previously identified in the first-line setting but have not been investigated when targeted therapies are administered after progression on immune therapy. We performed a retrospective single institution analysis of 40 metastatic melanoma patients receiving combined BRAF/MEK inhibitors after progression on an anti-PD-1 or ipilimumab plus nivolumab to assess response rate by RECIST 1.1, progression-free and overall survival (PFS and OS). Pretreatment clinical variables were analyzed for association with OS. Ipilimumab/nivolumab was the first-line immunotherapy regimen in 39 patients (97.5%), and BRAF^V600E/K^ mutations were present in 33 (83%) and 7 (17%) patients, respectively. The median OS from start of BRAF/MEK inhibitors was 20.3 months (1.73-106.4+, 95% CI of median 13.3-30.7). Clinical characteristics associated with worse survival prior to starting BRAF/MEK inhibitors included age > 60 years (median OS 14 vs. 28 months; HR 2.5; 95% CI 0.91-6.87, *P* = .023), ECOG-PS > 2 (median OS 7 vs. 33 months; HR 2.89; 95% CI 0.78-10.76, *P* = .018), and presence of bone metastases (median OS 9 vs. 52 months; HR 3.17; 95% CI 1.33-7.54, *P* = .002). These associations with shorter survival maintained their significance on multivariate analysis. If confirmed in larger cohorts, the identified prognostic variables can be used for stratification of patients in future randomized trials.

## Teaser

Long-term clinical outcomes for patients receiving BRAF/MEKi after progression on immune therapy have not been fully described, and clinical predictors of long-term response and survival are not known. This article reports an analysis of patients receiving BRAF/MEKi after progression on one or more lines of immune checkpoint inhibitor therapy to assess response and clinical variables associated with survival.

**Implications for Practice**

First-line treatment with immune checkpoint inhibitors improves survival outcomes compared to first-line therapy with combined BRAF/MEK inhibitors in metastatic melanoma containing BRAFV600 mutations. Clinical variables associated with outcome from treatment with combined BRAF/MEK inhibition were previously identified in the first-line setting but have not been investigated when targeted therapies are administered after progression on immune checkpoint inhibitors. We found that clinical characteristics associated with worse survival prior to starting BRAF/MEK inhibitors included age > 60 years (median OS 14 vs. 28 months; HR 2.5; 95% CI 0.91-6.87, *P* = .023), ECOG-PS > 2 (median OS 7 vs. 33 months; HR 2.89; 95% CI 0.78-10.76, *P* = .018), and presence of bone metastases (median OS 9 vs. 52 months; HR 3.17; 95% CI 1.33-7.54, *P* = .002). If confirmed in larger cohorts, the identified prognostic variables can be used for stratification of patients in future randomized trials.

## Background

The current standard of care for first-line treatment of metastatic melanoma includes the programmed cell death 1 (PD-1) inhibitors pembrolizumab or nivolumab as single agents, or combinations of nivolumab with either ipilimumab [anti-cytotoxic T lymphocyte-associated protein 4 (CTLA-4)] or relatlimab [anti lymphocyte activating gene-3 (LAG-3)].^1-7^ In addition, for the 40% of cutaneous melanoma patients whose tumors harbor BRAF^V600E/K^ mutations, 3 different combinations of targeted therapy against BRAF and MEK (BRAF/MEKi), including vemurafenib/cobimetinib, dabrafenib/trametinib, and encorafenib/binimetinib, and a combination of cobimetinib/vemurafenib with atezolizumab (anti-PD-L1), have been approved by regulatory authorities.^8-11^ Both checkpoint inhibitor immunotherapy and targeted therapy prolong progression-free and overall survival compared with chemotherapy in metastatic melanoma.

In patients with BRAF^V600^ mutations, prior phase II and phase III studies had shown that first-line treatment with anti-PD-1 alone or ipilimumab plus nivolumab (ipi/nivo) produced long-term objective responses that were maintained despite discontinuation of treatment. Moreover, cross-trial comparisons suggested that survival outcomes were better with first-line immune checkpoint inhibitors compared to first-line therapy with BRAF/MEKi.^12,13^ The latter observations were confirmed by the DREAMseq trial, which randomized 265 patients with newly diagnosed metastatic melanoma to the sequence of ipi/nivo followed upon disease progression by dabrafenib plus trametinib versus the inverse sequence, and showed that the 2-year overall survival (OS) rate was superior for those starting with ipi/nivo (71.8% vs. 51.5%; log-rank *P* = .010).^12,14^

Administration of BRAF/MEKi following disease progression on immune checkpoint inhibitors is a standard of care, although limited prospective data have been collected. Prior studies identified disease stage, baseline lactate dehydrogenase (LDH), and baseline Eastern Cooperative Oncology Group performance status (ECOG PS) as important factors predictive of outcome in metastatic melanoma treated with BRAF/MEKi in the first-line setting.^15-23^ Long-term clinical outcomes for patients receiving BRAF/MEKi after progression on immune therapy have not been fully described, and clinical predictors of long-term response and survival are not known and may be different than those already described for patients receiving targeted therapy as the first-line therapy for metastatic disease. Therefore, we performed a retrospective analysis of patients receiving BRAF/MEKi after progression on one or more lines of immune checkpoint inhibitor therapy to assess objective response rates, PFS and OS, and clinical variables associated with survival.

## Methods

### Patients

We retrospectively identified patients with metastatic melanoma treated at our institution from 2011 to 2020 who met the following inclusion criteria: BRAF^V600E/K^ mutation; had not received BRAF/MEKi before immune checkpoint inhibitors; and received ipi/nivo or anti-PD-1 (alone or in combination with other immune therapy agents) followed by BRAF/MEKi upon progression. We included patients who had received multiple lines of immune checkpoint inhibitors provided that at least one regimen of immune checkpoint inhibitors was administered before BRAF/MEKi. Patients were also included if the BRAF-MEKi regimen was combined with immune checkpoint inhibitors. There was no restriction on therapies administered upon progression on the initial BRAF/MEKi regimen.

### Clinical Characteristics and Endpoints

The following clinical characteristics prior to initiation of BRAF/MEKi were analyzed for potential association with treatment outcome: age, sex, type of BRAF mutation, LDH, ECOG performance status, sites of metastases, prior toxicity from immunotherapy, disease response to prior immunotherapy, duration of treatment on checkpoint inhibitor immunotherapy (>6 months versus < 6 months), and use of immunotherapy in addition to or upon progression on BRAF/MEKi. Clinical response to therapy was categorized as complete (CR), partial (PR), stable disease [SD] for more than 6 months, or progression of disease (PD) using RECIST 1.1 criteria, and outcome was analyzed by grouping CR + PR + SD > 6 months versus PD. An additional category of mixed response to immune checkpoint inhibitors (any site of tumor regression despite overall PD by RECIST 1.1 criteria) was also explored, and outcome was analyzed by grouping PD without mixed response versus CR/PR/SD > 6 months/PD with mixed response. Overall safety was assessed as adverse events of any grade occurring in more than 5% of patients per NCI CTCAEv5. Progression-free survival (PFS) and OS were calculated from the start date of BRAF/MEKi.

### Statistical Analyses

Clinical data were analyzed by descriptive statistics. Survival (PFS and OS) was analyzed by Cox regression and Kaplan-Meier method. Univariate analysis was calculated with log-rank (Mantel-Cox test) and multivariate analysis with Cox proportional hazards regression.

## Results

### Patient Characteristics

Forty patients were included in the analysis. One patient received a brief course of vemurafenib (<1 month due to toxicity) and was off therapy for 6 months before developing PD and starting on ipi/nivo and therefore was included. Patient characteristics and treatment are provided in Table 1. Eleven patients (27.5%) had distant metastases at the first diagnosis of melanoma. Among the 29 patients with a prior diagnosis of early-stage disease, 6 received adjuvant therapy [interferon (*n* = 4), nivolumab (*n* = 1), and ipilimumab (*n* = 1)]. Ipi/nivo was the first-line immunotherapy regimen in 39 (97.5%) patients. BRAF^V600E/K^ mutations were present in 33 (83%) and 7 (17%), respectively. Median patient age was 55 years (20-85), median ECOG-PS was 1 (0-4), and median LDH was 268 mg/dL (151–11 300). The most common sites of metastatic disease were lymph nodes (*n* = 30, 75%), lung/pleura (*n* = 24, 60%), skin/soft tissue (*n* = 22, 55%), liver (*n* = 19, 47.5%), bone (*n* = 16, 40%), brain (*n* = 15, 37.5%), bowel/peritoneum (*n* = 11, 27.5%), breast (*n* = 6, 15%), and pancreas or adrenal glands (*n* = 5 each, 12.5% each).

**Table 1.** Demographics and treatment data

Sex	Female (*n* = 24, 60%)
	Male (*n* = 16, 40%)
BRAF mutation	BRAF^V600E^ mutated (*n* = 33, 83%)
	BRAF^V600K^-mutated (*n* = 7, 17%)
Prior adjuvant therapy	Yes (*n* = 6, 21%*)
	Interferon (*n* = 4)
	Nivolumab (*n* = 1)
	Ipilimumab (*n* = 1)
	No (*n* = 23, 79%*)
	*From the total of M0 patients (*n* = 29)
First-line immunotherapy regimen	Ipilimumab + nivolumab (*n* = 39, 97.5%) Anti-PD1 alone (*n* = 1, 2.5%)
Median duration of first-line immunotherapy	3.5 months (0.5-42.8)
Best response to immunotherapy prior to BRAF/MEKi	CR (*n* = 2, 5%)
	PR (*n* = 9, 22.5%)
	SD > 6 months (*n* = 1, 2.5%)
	PD with mixed response (*n* = 9, 22.5%)
	PD without mixed response (*n* = 18, 45%)
	Not available (*n* = 1, 2.5%)
Median age	55 years (20-85)
Median ECOG-PS	1 (0-4)
Median LDH prior to BRAF/MEKi	268 mg/dL (151–11 300)
Sites of metastatic disease	Lymph nodes (*n* = 30, 75%)
	Lung/pleura (*n* = 24, 60%)
	Skin/soft tissue (*n* = 22, 55%)
	Liver (*n* = 19, 47.5%)
	Bone (*n* = 16, 40%)
	Brain (*n* = 15, 37.5%)
	Bowel/peritoneum (*n* = 11, 27.5%)
	Breast (*n* = 6, 15%)
	Pancreas (*n* = 5, 12.5%)
	Adrenal (*n* = 5, 12.5%)
BRAF/MEKi regimens	Dabrafenib plus trametinib (*n* = 35, 87.5%) Binimetinib plus encorafenib (*n* = 5, 12.5%).
Median duration of BRAF/MEKi therapy	7 months (0.5–106 + months)
Best response to BRAF/MEKi	CR (*n* = 4, 10%)
	PR (*n* = 26, 65%)
	SD > 6 months (*n* = 4, 10%)
	PD (*n* = 6, 15%)
Subsequent immune checkpoint inhibition in addition to or upon progression on BRAF/MEKi	Yes (*n* = 26, 65%)
	No (*n* = 14, 35%)

Abbreviations: BRAF/MEKi: BRAF plus MEK inhibitors; PD1: programmed cell death 1; LDH: lactate dehydrogenase; ECOG-PS: Eastern Cooperative Oncology Group performance status.

Median duration of first-line immunotherapy was 3.5 months (0.5-42.8 months). Best response to first-line immunotherapy was CR (*n* = 2, 5%), PR (*n* = 9, 22.5%), SD > 6 months (*n* = 1, 2.5%), PD with mixed response (*n* = 9, 22.5%), PD without mixed response (*n* = 18, 45%), and one patient was not evaluable for response (*n* = 1, 2.5%). Most patients received subsequent immune checkpoint inhibition in addition to or upon progression on BRAF/MEKi (*n* = 26, 65%).

### Treatment Outcomes

Median follow-up for all patients was 33 months (3–172 + months). The median duration of BRAF/MEKi therapy was 7 months (0.5-106 months). The best response by RECIST criteria to BRAF/MEKi was CR (*n* = 4, 10%), PR (*n* = 26, 65%), SD > 6 months (*n* = 4, 10%), and PD (*n* = 6, 15%).

**Figure 1.**
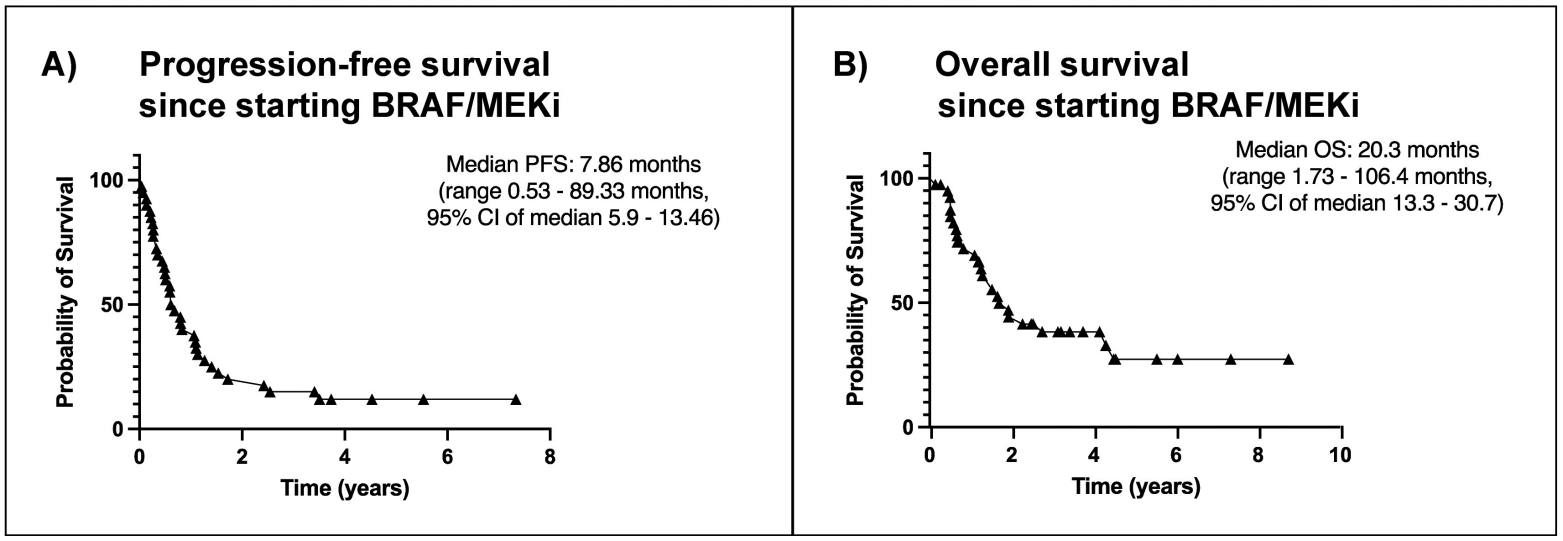
Progression-free survival (**A**) and overall (**B**) since starting BRAF/MEKi. Abbreviations: BRAF/MEKi: BRAF plus MEK inhibitors; PFS: progression-free survival; OS: overall survival.

After starting BRAF/MEKi, median PFS was 7.86 months (0.53-89.33, 95% CI 5.9-13.46), Fig. 1, with a 1-year PFS of 37.5% and 2-year PFS of 17.5%. Median overall survival was 20.3 months (1.73-106.4, 95% CI 13.3-30.7), with a 1-year OS of 69% and 2-year OS of 41.5%.

### Analysis of Clinical Variables for Association With OS

Prior to BRAF/MEKi, age > 60 years (median OS 14 vs. 28 months, HR 2.5; 95% CI 0.91-6.87, *P* = .023), ECOG-PS 3-4 (median OS 7 vs. 33 months; HR 2.89; 95% CI 0.78-10.76, *P* = .018), and presence of bone metastases (median OS 9 vs. 52 months, HR 3.17; 95% CI 1.33-7.54, *P* = .002) were associated with shorter OS on univariate analysis. Other baseline variables including sex, LDH prior to BRAF/MEKi, M stage (M1a/M1b versus M1c/M1d), number of organ metastatic sites (≥3 versus < 3), sites of metastatic disease (skin/lymph nodes/soft tissue, lung, liver, brain, bowel/peritoneum), toxicity to prior immunotherapy, length of time between start of immunotherapy and BRAF/MEKi (≥6 months vs < 6 months), and best response to immunotherapy (PD without mixed response vs CR/PR/SD > 6 months/PD with mixed response), and on treatment variables such as best response to BRAF/MEKi (PD vs CR/PR/SD), immunotherapy in addition to or upon progression to BRAF/MEKi, and toxicity to BRAF/MEKi were not statistically associated with OS, Table 2.

**Table 2.** Univariate analysis of survival using log-rank (Mantel-Cox test)

Variables (number of patients)		Median survival (months)	HR (95% CI)	*P*-value
Baseline variables				
Age prior to BRAF/MEKi	≥60 years (10) vs. <60 years (30)	14 vs. 28	2.5 (0.91-6.87)	.023
Sex	Female (24) vs. male (16)	21 vs. 52	1.18 (0.52-2.68)	.696
LDH prior to BRAF/MEKi	Above ULN (25) vs. normal (12)	16 vs. not reached	2.38 (1.03-5.48)	.074
ECOG-PS prior to BRAF/MEKi	3-4 (7) vs. 0-2 (30)	7 vs. 33	2.89 (0.78-10.76)	.018
Best response to immunotherapy	PD without mixed response (18) vs. CR/PR/SD/PD with mixed response (21)	15 vs. 44	1.26 (0.93-4.78)	.062
Toxicity to immunotherapy	Yes (29) vs. no (11)	26 vs. 5	0.58 (0.22-1.50)	.262
Length of time between start of immunotherapy and start of BRAF/MEKi	Less than 6 months (20) vs. 6 months or more (20)	18 vs. 23	0.81 (0.37-1.80)	.613
M stage	M1c/M1d (32) vs. M1a/M1b (8)	18 vs. 52	2.75 (1.14-6.63)	.084
Skin, LN, soft tissue	Yes (36) vs. no (4)	23 vs. 17	0.86 (0.24-3.10)	.810
Lung	Yes (24) vs. no (16)	23 vs. 21	0.96 (0.44-2.13)	.927
Liver	Yes (19) vs. no (21)	16 vs. 33	1.92 (0.86-4.23)	.095
Bone	Yes (16) vs. no (24)	9 vs. 52	3.17 (1.33-7.54)	.002
Brain	Yes (15) vs. no (25)	28 vs. 18	0.77 (0.35-1.71)	.531
Bowel/peritoneum	Yes (11) vs. no (29)	21 vs. 23	1.04 (0.43-2.51)	.927
Number of metastatic sites prior to BRAF/MEKi	≥3 (14) vs. <3 (26)	20 vs. 27	1.24 (0.55-2.80)	.615
On treatment variables				
Best response to BRAF/MEKi	PD (6) vs. CR/PR/SD (34)	5 vs. 26	2.05 (0.58-7.23)	.128
Immunotherapy in addition to or upon progression to BRAF/MEKi	Yes (26) vs. no (14)	23 vs. 18	0.99 (0.43-2.30)	.981
Toxicity to BRAF/MEKi	Yes (31) vs. no (9)	26 vs. 7	0.59 (0.21-1.61)	.211

Abbreviations: BRAF/MEKi: BRAF plus MEK inhibitors; HR: hazard ratio; LDH: lactate dehydrogenase; ECOG-PS: Eastern Cooperative Oncology Group performance status; ULN: upper limit of normal; LN: lymph nodes.

On multivariate analyses, the same variables were confirmed to be independently associated with shorter OS [older age (HR 3.97; 95% CI 1.50–10.47, *P* = .005); higher ECOG-PS (HR 3.19; 95% CI 1.05–8.81, *P* = .029), and presence of bone metastases (HR 4.81; 95% CI 1.92–12.89, *P* = .001)], Table 3.

**Table 3.** Multivariable analysis using Cox proportional hazards regression

Variables	HR (95% CI)	*P*-value
Age prior to BRAF/MEKi > 60 years	3.97 (1.50-10.47)	.005
ECOG prior to BRAF/MEKi 3-4	3.19 (1.05-8.81)	.029
Bone metastases	4.81 (1.92-12.89)	.001

Abbreviations: BRAF/MEKi: BRAF plus MEK inhibitors; HR: hazard ratio; ECOG-PS: Eastern Cooperative Oncology Group performance status.

We analyzed 2 additional subgroups of patients with exceptional outcomes as listed in Supplementary Table S1. The first subgroup included patients who were alive for ≥4 years from start of BRAF/MEKi (*n* = 8/40, 20%) and the second subgroup included patients whose disease did not progress despite stopping BRAF/MEKi due to toxicity or patient choice and were still alive at last follow-up (*n* = 4/40, 10%). Two out of 4 of the latter patients from the second subgroup had follow-up ≥4 years and are included among the 8 patients in the first subgroup. The clinical characteristics of the ≥4 years long-term survivors were similar to the overall population. For these 8 long-term survivors, the median duration of first-line immunotherapy was 3.5 months (0.5-42.8 months) and all patients had PD as best response to immunotherapy. Five of the 8 were considered to have had mixed response to immune checkpoint inhibitors by treating physicians based on clinical and radiological findings. All had BRAF^V600E^ mutations, and the median duration of second-line BRAF/MEKi was 29.7 months (5.4-55.1 months). Seven had best response of PR to BRAF/MEKi. All but one received immune checkpoint inhibition in addition to or upon progression on BRAF/MEKi.

From the second subgroup of 4 patients who stopped BRAF/MEKi due to toxicity or patient choice but maintained disease control without further therapy and remained alive at last follow up, all started with ipi/nivo and median duration of first-line immunotherapy was 25 months (16-42 months). Two had PR as best response to immunotherapy while the remaining 2 had PD by RECIST criteria, but with a mixed response. Median duration of BRAF/MEKi therapy was 3.5 months (0.5-16 months) and best response to BRAF/MEKi was PR in 3 and CR in one. One patient received concomitant immune checkpoint inhibition with the BRAF/MEKi regimen.

### Safety

Adverse events during prior treatment with immune checkpoint inhibitors and during subsequent BRAF/MEKi are listed in Table 4. Most common adverse events of any grade (occurring in more than 5% of patients) from immune checkpoint inhibitors were fatigue (*n* = 5, 12.5%), rash (*n* = 3, 7.5%), hepatitis (*n* = 3, 7.5%), arthralgia (*n* = 3, 7.5%), diarrhea (*n* = 3, 7.5%), fever (*n* = 3, 7.5%), nausea (*n* = 2, 5%), vomiting (*n* = 2, 5%), and neuropathy (*n* = 2, 5%). The most common adverse events of any grade (occurring in more than 5% of patients) from BRAF/MEKi were fever (*n* = 20, 50%), nausea (*n* = 9, 22.5%), fatigue (*n* = 6, 15%), diarrhea (*n* = 5, 12.5%), vomiting (*n* = 5, 12.5%), and arthralgia (*n* = 2, 5%).

**Table 4.** Safety

Most common adverse events of any grade (occurring in more than 5% of patients) after first-line immune checkpoint inhibitors	
Fatigue	*n* = 5, 12.5%
Rash	*n* = 3, 7.5%
Hepatitis	*n* = 3, 7.5%
Arthralgia	*n* = 3, 7.5%
Diarrhea	*n* = 3, 7.5%
Fever	*n* = 3, 7.5%
Nausea	*n* = 2, 5%
Vomiting	*n* = 2, 5%
Neuropathy	*n* = 2, 5%
Most common adverse events of any grade (occurring in more than 5% of patients) after second-line BRAF/MEKi	
Fever	*n* = 20, 50%
Nausea	*n* = 9, 22.5%
Fatigue	*n* = 6, 15%
Diarrhea	*n* = 5, 12.5%
Vomiting	*n* = 5, 12.5%
Arthralgia	*n* = 2, 5%

Abbreviation: BRAF/MEKi: BRAF plus MEK inhibitors.

## Discussion

This was a retrospective study assessing the clinical activity, outcomes, and potential clinical factors associated with survival among patients with BRAF^V600^-mutated metastatic melanoma treated with BRAF/MEKi after disease progression on immune checkpoint inhibition. The median follow-up of 33 months in our cohort also provided the first opportunity to assess the potential long-term benefit of targeted therapy as administered in a standard of care setting to patients no longer responsive or resistant to doublet immune checkpoint therapy.

Limited prospective data are available for patients who received BRAF/MEKi after progression on immune checkpoint inhibitors, particularly combined ipilimumab plus nivolumab. At the time of publication of the DreamSEQ trial, 44 patients developed disease progression from first-line therapy with ipilimumab plus nivolumab, and only 21 of the latter were registered to step 2 of the study for treatment with second-line BRAF/MEKi.^12^ Response data were not provided for second-line targeted therapy. In the SECOMBIT trial, 38 patients receiving first-line treatment with ipilimumab + nivolumab received BRAF/MEKi at the time of progression.^13^ The objective response rate to second-line BRAF/MEKi was 57.9%, similar to the response rate of 75% observed in our cohort of 40 patients. However, at the time of publication, the SECOMBIT trial did not report duration of response, progression-free or overall survival from initiation of second-line targeted therapy.

We observed that treatment with BRAF/MEKi following progression on immune therapy, including at least one regimen of ipilimumab plus nivolumab, is associated with a 2-year survival of 41.5%, and a 4-year survival rate of 20%. Ten percent of our cohort were able to discontinue BRAF/MEKi treatment without subsequent disease recurrence. This observed level of activity is indicative of meaningful clinical benefit in at least a subset of patients. The potential for long-term survival with second-line BRAF/MEKi should be considered by patients and physicians when choosing other potential treatment options in this setting including clinical trials or, if approved in the future, adoptive cell therapy with tumor-infiltrating lymphocytes (TIL).^24^ Nevertheless, the 2-year PFS rate was only 17.5%, clearly indicating the need to develop more effective therapies.

Our data showed that age of 60 years or older, ECOG-PS 3-4, and presence of bone metastases were independently associated with worse survival outcomes as per multivariate analysis. Clinical variables associated with worse outcome post-immune checkpoint inhibitors appeared to be different from those identified in a large pooled 3-year analysis of BRAF inhibitor-naïve patients treated with dabrafenib plus trametinib across phase II (BRF113220, part C) and phase III (COMBI-d, COMBI-v) registration trials. In the latter analyses, baseline LDH and number of organ sites with metastases were strongly associated with worse PFS and OS, and the baseline sum of lesion diameters was identified as a predictor of progression.^18,20^

The DREAMseq trial explored prognostic features such as age (<40, 40-60, >60), ECOG-PS (0 or 1), sex, type of BRAF mutation, LDH, M stage (<M1c or M1c), and a combination of favorable features (ECOG-PS 0 plus normal LDH and <M1c) and concluded that in all of these subtypes, the strategy of starting with ipi/nivo followed by BRAF/MEKi was numerically associated with improved survival.^12,14^ There is as of yet no reported results from this study on features of long-term survivors from the start of BRAF-MEKi in the second-line setting.

Despite progression on immunotherapy, a subset of patients receiving BRAF/MEKi have prolonged OS, including some that maintain durable responses even after discontinuing BRAF/MEKi. Presumed immune-related side effects occurring during targeted therapy have been associated with longer clinical benefit.^25-27^ We also hypothesized that patients demonstrating tumor regression from prior immune therapy, despite ultimate overall disease progression, may have better outcomes from second-line targeted therapy. In addition to reducing tumor burden, BRAF/MEKi potentially modulate the immunosuppressive tumor microenvironment including antigen presentation, secretion of cytokines, and the ratio of suppressive versus effector tumor-infiltrating lymphocytes in BRAF^V600^-mutated tumors.^25,28,29^ We did not find that adverse events were associated with better survival, including toxicity from prior immunotherapy, or toxicity during BRAF/MEKi treatment. There was also no association of OS with length of time on immunotherapy prior to BRAF/MEKi, prior tumor regression from immune therapy, or use of immune checkpoint inhibitors concomitantly or upon progression on BRAF/MEKi.

Our 8 patients (20%) with very long survival of more than 4 years from start of BRAF/MEKi had numerically similar clinical characteristics when compared to the remaining patients, apart from longer duration of BRAF/MEKi therapy, the latter being expected as they lived longer. Interestingly, our 4 patients (10% of the entire cohort) who still maintained disease control despite stopping BRAF/MEKi due to toxicity or patient choice also had similar overall clinical characteristics but were notably on immunotherapy for a numerically longer median time than the whole population (25 months versus 3.5 months), and shorter median time on BRAF/MEKi (3.5 months versus 7 months), although the small sample size precludes definitive conclusions.

In summary, we found that BRAF/MEKi following progression on immune checkpoint inhibitors is associated with high response rates and a small number of long-term survivors. We did not identify clinical characteristics that could identify these long-term survivors prior to treatment. However, age > 60 years, ECOG-PS > 2 and presence of bone metastases were independently associated with worse survival outcomes in our population. We acknowledge that there are several limitations to our study due its retrospective single-center nature without prospective controlled radiologic and clinical data collection, which may bias our results. We did not report the data on additional administration of local therapies, such as gamma-knife radiation therapy to brain metastases, stereotactic body radiation therapy to systemic metastases and/or local resection of brain and/or systemic metastases, which in certain cases allowed continued therapy beyond the date of first documented PD.

Outcomes on BRAF/MEKi after immune therapy remain suboptimal, and strategies to improve duration of response and survival in these patients will likely require addition of agents to BRAF/MEKi. The potential benefit of any combination with BRAF/MEKi would need to be confirmed in randomized trials. Our data, if confirmed in larger cohorts, would guide stratification for future phase III trials in this setting. Further exploration of biomarkers is still needed as a basis for improved treatment decisions on an individual basis, which is crucial to minimize toxicity and increase treatment efficacy and survival.

## Supplementary Material

Supplementary material is available at *The Oncologist* online.

oyad300_suppl_Supplementary_Tables_1

## Data Availability

The datasets generated during and/or analyzed during the current study are available from the corresponding author on reasonable request.
