# Supplementary material for: Clinical Predictors of Survival in Patients With BRAFV600-Mutated Metastatic Melanoma Treated With Combined BRAF and MEK Inhibitors After Immune Checkpoint Inhibitors
Source: Oncologist. 2023 Nov 16;29(4):e507–13. doi: 10.1093/oncolo/oyad300 (PMC10994263; doi:10.1093/oncolo/oyad300)
Supplement: oyad300_suppl_Supplementary_Tables_1 [file oyad300_suppl_supplementary_tables_1.docx]

| **Supplementary table 1.** Additional subgroups of patients with exceptional outcomes | | | | | | | | | | | | | | | | |
| --- | --- | --- | --- | --- | --- | --- | --- | --- | --- | --- | --- | --- | --- | --- | --- | --- |
| Subgroup | Survival status | Prior immune therapy | Survival status | Length of prior immune therapy (months) | Best response to prior immune therapy | OS (months) | PFS  (months) | LDH | ECOG PS | Number of organs with metastatic involvement | Length of BRAF-MEKi therapy  (months) | Concurrent Rx with ICI | Stopped BRAF/MEKi | Time since stopping BRAF/MEKi without progression (months) | Time since stopping all therapy without progression | Therapies subsequent to BRAF/MEKi |
| 1 | alive | Ipi/nivo | alive | 1 | PD | 106 | 31 | 158 | 0 | 2 | 7 | No | no | NA | NA | Clinical trial), ipi/nivo, dab+tram |
| 1 | alive | Ipi/nivo | alive | 4 | MR | 55 | 55 | 262 | 0 | 4 | 55 | No | no | NA | NA | NA |
| 1 | alive | Ipi/nivo | alive | 4 | MR | 50 | 21 | 535 | 0 | 4 | 50 | No | no | NA | NA | NA |
| 1 | alive | Pembrolizumab | alive | 1 | PD | 74 | 43 | 258 | 1 | 2 | 43 | Yes | yes | 31 | NA | Nivo |
| 1 | dead | Ipi/nivo | dead | 1 | MR | 55 | 13 | 327 | 2 | 6 | 10 | Yes | yes | 45 | NA | Clinical trial |
| 1 | dead | Ipi/nivo | dead | 3 | PD | 57 | 19 | 268 | 2 | 2 | 47 | No | yes | 5 | NA | Nivo |
| 1,2 | alive | Ipi/nivo | alive | 16 | MR | 67 | 67 | 151 | 3 | 3 | 5 | Yes | yes | 62 | 62 | None |
| 1,2 | alive | Ipi/nivo | alive | 43 | MR | 89 | 89 | 216 | 0 | 8 | 16 | No | yes | 72 | 72 | None |
| 2 | alive | Ipi/nivo | alive | 20 | PR | 42 | 42 | 264 | 1 | 3 | 37 | No | yes | 5 | 5 | None |
| 2 | alive | Ipi/nivo | dead | 30 | PR | 5 | 3 | 241 | 1 | 4 | 2 | No | yes | 1 | 1 | None |

Subtitles: Subgroup 1: patients who were alive for > 4 years from start of BRAF/MEKi (n= 8/40, 20%); Subgroup 2: patients whose disease did not progress despite stopping BRAF/MEKi due to toxicity or patient choice and were still alive at last follow-up; Abbreviations: OS: overall survival; PFS: progression-free survival; LDH: lactate dehydrogenase; ECOG-PS: Eastern Cooperative Oncology Group performance status
